# antaRNA – Multi-objective inverse folding of pseudoknot RNA using ant-colony optimization

**DOI:** 10.1186/s12859-015-0815-6

**Published:** 2015-11-18

**Authors:** Robert Kleinkauf, Torsten Houwaart, Rolf Backofen, Martin Mann

**Affiliations:** 1 Bioinformatics Group, Department of Computer Science, University of Freiburg, Georges-Köhler-Allee 106, Freiburg, 79110 Germany; 2 Center for Biological Signaling Studies (BIOSS), University of Freiburg, Freiburg, Germany; 3 Center for Biological Systems Analysis (ZBSA), University of Freiburg, Freiburg, Germany; 4 Center for non-coding RNA in Technology and Health, University of Copenhagen, Grønnegårdsvej 3, Frederiksberg C, 1870 Denmark

**Keywords:** Pseudoknot RNA, Inverse folding RNA, RNAdesign, Synthetic biology, Biotechnology

## Abstract

**Background:**

Many functional RNA molecules fold into pseudoknot structures, which are often essential for the formation of an RNA’s 3D structure. Currently the design of RNA molecules, which fold into a specific structure (known as RNA inverse folding) within biotechnological applications, is lacking the feature of incorporating pseudoknot structures into the design. Hairpin-(H)- and kissing hairpin-(K)-type pseudoknots cover a wide range of biologically functional pseudoknots and can be represented on a secondary structure level.

**Results:**

The RNA inverse folding program antaRNA, which takes secondary structure, target GC-content and sequence constraints as input, is extended to provide solutions for such H- and K-type pseudoknotted secondary structure constraint.

We demonstrate the easy and flexible interchangeability of modules within the antaRNA framework by incorporating pKiss as structure prediction tool capable of predicting the mentioned pseudoknot types. The performance of the approach is demonstrated on a subset of the Pseudobase ++ dataset.

**Conclusions:**

This new service is available via a standalone version and is also part of the Freiburg RNA Tools webservice. Furthermore, antaRNA is available in Galaxy and is part of the RNA-workbench Docker image.

## Background

The recent years have seen an explosion in the discovery of non-coding RNAs associated with many different and surprising functions. Non-coding RNAs are involved in most regulatory processes, e.g. via interactions with proteins and other nucleotide sequences (DNA and RNA), or act as protein assembly platforms for complex ribonucleic particles. Due to this versatility, RNA molecules are now an emerging focus in synthetic biology and biotechnology. Aptamers against virtually any larger cellular molecule or even complete cells can be identified by SELEX RNA enrichment [1, 2]. This technique enables new molecular-medical applications for diagnostics and therapy [3–5] and the development of artificial biomaterials [6]. Another example is the procaryotic RNA-based CRISPR/Cas ‘immune system’ [7], which revolutionized genome editing [8].

By combining different functional RNA molecules in synthetic biology and biotechnological applications, synthetic constructs can be designed with a completely new functionality [9, 10]. However, the problem of compatibility occurs. In contrast to protein domains, functional RNAs are not easily fusable in a single new molecule since they mutually influence their structure. For that reason, one needs computational design tools as an important step in generating candidates for further testing. Since the function of an RNA is related to both sequence and the associated structure, we need to solve the problem of finding a sequence (under certain constraints) that folds into a functional structure. This is known as the inverse folding problem.

Among published approaches, different strategies have been pursued: Initial implementations realize simple sampling and local optimization techniques. For example, *RNAinverse* [11] samples sequences with subsequent local optimization, which was extended in *INFORNA* [12] with an improved seeding of the sequences. Newer algorithms mimic evolutionary processes (*fRNAkenstein* [13] and *ERD* [14]), but also show sophisticated improved optimization mechanics such as efficient ensemble defect optimization (*NUPACK* [15]) or fragment-based bonification methods (*RNAfbinv* [16]).

All above tools consider only nested secondary structures as design target. However, the functional structure usually involves crossing base pairs, forming so-called pseudoknots, that stabilize the tertiary three-dimensional structure [17, 18]. In addition, pseudoknots have been shown to be of high importance in specific functionality of respective RNA families, e.g. shown in human telomerase [19]. For example, the RFAM database (v12.0 07/2014) [20] lists 2,450 different RNA families with a wide variety of biological functions, of which 170 families are tagged with the annotation ‘pseudoknot’.

To our knowledge, so far the tools *Inv* [21] and *MODENA* [22] are the only approaches that allow for the design of sequences that fold into a given structure with pseudoknot features. *Inv* performs local optimization based on the loop decomposition of the target structure. *MODENA* uses a genetic heuristic to produce solution sequences, which are evaluated by applying either *IPknot* [23] and *hotknots* [24] as structure prediction programs. However, both tools lack the possibility to specify a targeted GC-content, which is an important requirement in practical design applications. The reason is simply that the GC-content of an RNA molecule can influence the efficiency of inherent functionality dramatically [25–27].

In [28], we have presented *antaRNA*, a sequence design tool that heeds the objectives formulated above. Within this paper, we present the extension of *antaRNA* for targeting pseudoknot structures. The tool provides the user with sequences that form the targeted (pseudoknot) structure as their minimum free energy (mfe) structure with a specified GC-content. For mfe-optimization, *pKiss* [29] is incorporated. Extending the already available constraint palette of *antaRNA*, soft sequence and soft fuzzy structure constraints are introduced. We present the parameter optimization for *pKiss* usage and compare *antaRNA*’s performance with *MODENA*. Furthermore, we emphasize *antaRNA*’s availability within the Freiburg RNA Tools webservice [30] for ad hoc usage using *forna* [31] for structure visualizations. In addition, *antaRNA* is embedded into a Galaxy-RNA-workbench Docker Image [32] for local large scale experiments.

## Implementation of antaRNA

In the following, we give a brief overview of *antaRNA*’s optimization approach. A detailed description including all formalisms is provided in [28]. Subsequently, we introduce the recent extension of *antaRNA* to the design of sequences for crossing pseudoknot structures.

### Overview

Given an RNA secondary structure constraint in extended dot-bracket notation $\mathbb {C}^{\text {str}}$, a targeted GC-content value $\mathbb {C}^{\text {gc}}$ and supplemental sequence constraint $\mathbb {C}^{\text {seq}}$ using IUPAC nucleotide definitions, *antaRNA* [28] solves the RNA inverse fold problem.

**Fig. 1:**
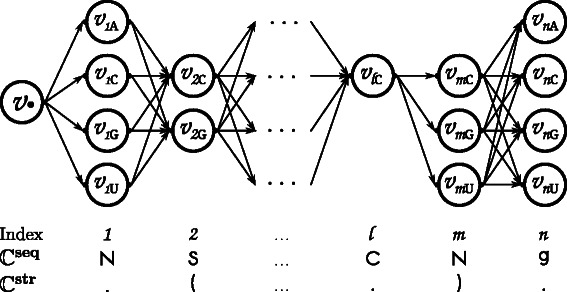
Schematic Terrain Graph Starting from a vertice *v*_∙_, n subsequent vertices within the graph are visited by a single ant during its walk through the terrain in order to assemble an RNA sequence. For each visited vertex, a corresponding nucleotide information is incorporated to the corresponding position within the sequence. The specific vertex of position *j* is chosen probabilistic according to the set of edges leading away of the current vertex *i*. Hereby specific pheromone and terrain contributions of the edges influence the probabilities per position. The interplay of an increasing sequence constraint specificity and the applied inductive structure constraint determine the number of vertices for some sequence positions. For example, even though position *m* is labeled with an ‘N’ as sequence constraint, the position only has three vertices due to the sequence constraint of position *2* and its request to form a base pair with the nucleotide at position *m*. This leads to the removal of the ‘A’ nucleotide vertex in *m*, since this cannot base pair with neither ‘C’ nor ‘G’ at position *2*

To this end, the Ant Colony Optimization technique [33, 34], an automatically adapting local search scheme, is applied. It mimics the ants’ adaptive search for food within a given terrain (see Fig. 1 and Algorithm 1). Here, the terrain is a graph encoding of the inverse folding problem with weighted edges representing the ants’ pheromone that guides their search. During an ant’s walk, the current pheromonic state of the terrain guides an ant to make its decisions in selecting certain edges, which lead to nucleotide-emitting vertices. Within one walk, an ant assembles a solution sequence. Dependent on the quality of the sequence with respect to its structure, sequence and GC-distances to the respective constraints, the pheromonic state of the terrain graph is updated according to a solution’s quality score.

Therefore, after a certain number of consecutive sequence assemblies and terrain adaptations, the features of the assembled sequences converge towards the anticipated constraints of the input [28].

### Pseudoknot structures

A main focus of inverse folding is the probability that the designed sequences fold into a given target structure. To this end, for each assembled sequence the minimum free energy (mfe) structure is predicted. *antaRNA*’s structural distance measure, *d*_str_, evaluates the compliance of an mfe structure with the structural target. This distance guides the pheromone update of the terrain.

For nested target structures, mfe prediciton was done using *RNAfold* from the *ViennaRNA*-package [11, 35]. In this work, structure constraints have been extended to support crossing, i.e. pseudoknot, structures. To this end, the structure predictor employed in *antaRNA* was substituted with the program *pKiss* [29]. *pKiss* is capable of predicting two specific subclasses of pseudoknots: hairpin (H-type) and kissing hairpin (K-type) structures. Both types are biologically important, even though H-type pseudoknots have been reported more often in the literature and in data bases. Both play crucial roles in various key functional domains of RNAs [36].

Since mfe structure prediction is done for each assembled sequence, its time complexity is of importance. *RNAfold* finds nested structures with a time complexity of $\mathcal {O}(n^{3})$ for sequences of length *n* [37]. *pKiss* predicts mfe structures with pseudoknots in $\mathcal {O}(n^{4})$ when heuristics are applied. For exact mfe calculations, *pKiss* requires $\mathcal {O}(n^{6})$ time [29]. *antaRNA* provides the possibility to choose the prediction method applied by *pKiss*.

*antaRNA* was extended such that the structure parsing and management now respects the increased complexity of pseudoknotted structures. The allowed set of brackets within the dot-bracket structure constraint notation was extended to “()[]{}<>” as it is used by *pKiss*. Furthermore, a *pKiss*-optimized set of parameters for *antaRNA* has been identified, when using *pKiss* for structure prediction. This is discussed in the following sections.



### New features

In addition to pseudoknot structure support, *antaRNA* now provides soft sequence and improved hard fuzzy structure constraint definitions. Both increase the level of detail, at which the target constraints can be defined.

The *soft sequence constraint* now allows to specify (in lower case letters) the preference for a nucleotide at a certain position. The nucleotide is then not enforced but penalized in the sequence quality assessment if a different nucleotide was set. This enables more flexibility to the *antaRNA*-based sequence design.

The fuzzy structure constraint, based on the already existent implicit block constraint framework of *antaRNA* [28], allows to define regions of structural interaction (using lower case letters), in which no explicit structure is predefined. For instance, the structural constraint $\mathbb {C}^{\text {str}} = $‘(aaaaaa)’ is neither violated if a base pair is present in the a-block, e.g. ‘((....))’ or ‘(.(...))’, nor if no base pair is designed, i.e. ‘(......)’. So far, if no base pair was formed within such a block no penalty (structural distance) was applied. By introducing the new *hard fuzzy structure constraint* framework (encoded by upper case letters), now the ‘no base pair’ case is penalized, if found within a solution. The structural distance is increased by the equivalence of one missing explicit base pair for each upper case block that shows no base pair. Therefore, at least one base pair has to be designed within a defined *hard fuzzy structure constraint* block. The latter adds a more imperative form of fuzziness to the structure constraint definition within *antaRNA*.

## Parameter optimization and benchmarking

*antaRNA* was extended such that the usage of *pKiss* as a structure prediction program is possible. *pKiss* optionally replaces *RNAfold* as the mfe prediction tool. This made it necessary to identify a new set of *pKiss*-specific parameters for the *antaRNA* pipeline. In the following, we provide details about the data used for parameter optimization and the benchmarking of different design tools.

### Data

**Fig. 2:**
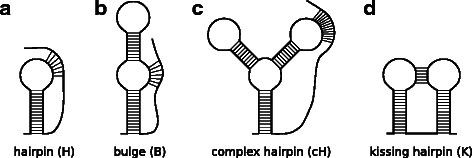
Pseudoknot Types **a** regular simple hairpin pseudoknot (H), **b** bulge hairpin pseudoknot (B), **c** complex hairpin pseudoknot (cH), **d** kissing hairpin pseudoknot (K). The complexity order is H <*B*<*cH*<K. Technically B-type and cH-type are more complex forms of H-type pseudoknots

The parameter optimization and benchmarking of *antaRNA* was performed on partitions of the pseudoknot specific PseudoBase ++ database [38] (download of 2014/12). PseudoBase ++ contains 304 entries. 37 entries, which did not show canonical base-pairings (AU,GC,GU), were excluded from the dataset. Pseudoknot structures can be grouped into types according to their composition. Figure 2 depicts regular simple hairpin pseudoknot (H), bulge hairpin pseudoknot (B), complex hairpin pseudoknot (cH), and kissing hairpin pseudoknot (K). *pKiss* supports H- and K-type pseudoknots, where B- and cH-type pseudoknots are subvariants of the H-type. We excluded further 2 entries from the derived PseudoBase ++ dataset, that did not fall into these classes.

From this pool of 265 structural constraints, the training set was derived. We selected at random 16 entries of increasing lengths, while each entry has a minimal length difference of 5 to the next shorter or longer structure. The final training set consists of 7 H-type and 3 B-type as well as 6 cH-type structures of higher complexity e.g. due to additional multiloops.

The remaining 249 instances were pooled into the test set. It contains 209 H-type, 29 B-type, 8 cH-type and 3 K-type structures. The datasets are available on the tool’s web page.

### Parameter optimization

The parameter optimization used a grid search for the best set of *pKiss*-specific parameters. For each tested parameter combination, designs for the structures from the training set and target GC values $\mathbb {C}^{\text {gc}}\in \{0.25,0.5,0.75\}$ were evaluated. Per produced sequence design, a time limitation of 600 seconds was applied.

The parameter set showing the highest average design quality was selected as default parameter set and is used in the following. For a detailed listing of the optimized parameters please refer to the tool’s web site. Design quality covers the achieved GC deviation, the reached structural deviations and the consumed runtime. For details concerning design quality evaluation see [28].

### Benchmark

The performance of *antaRNA* for the design of sequences folding into pseudoknot structures was benchmarked on the test data. For each structure constraint $\mathbb {C}^{\text {str}}$ in the test set, ten sequence designs were done for three different target $\mathbb {C}^{\text {gc}}$ values (0.25,0.5,0.75), resulting in 7,470 design experiments. Per experiment the runtime was restricted to 1,200 seconds.

The benchmarking of *MODENA* was performed on the same test data and has been kindly provided by the authors of *MODENA*. It was benchmarked for both structure prediction methods supported, namely *IPknot* and *hotknots*. Since it does not support GC-content constraints, no target GC-value was set.

The benchmark was evaluated based on the structural distances of a sequence’s mfe structure to the respective structure constraint and the deviation of the GC-content from the target value. For *MODENA*, no special GC target constraint was specified and thus the achieved GC-value was assessed. A comparison towards the performance of the described program *Inv* was not possible due to its unavailability.

## Results

**Fig. 3:**
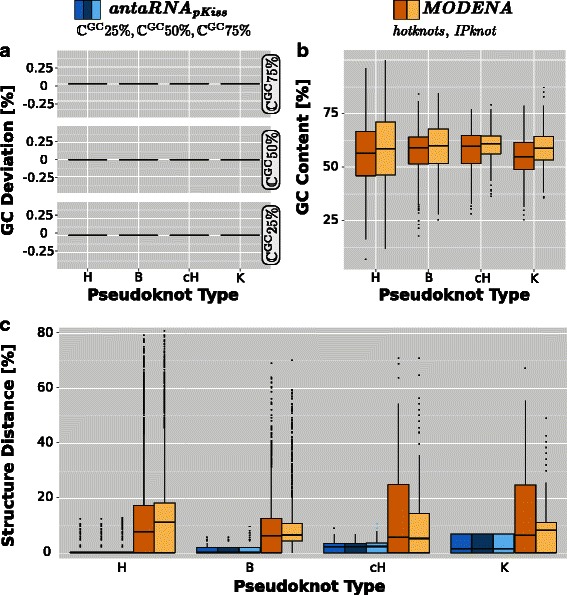
Constraint Compliance for Pseudoknot Categories. **a** GC-deviation of *antaRNA* for different $\mathbb {C}^{\text {gc}}$, **b** Intrinsic GC values of *MODENA* using *hotknots* (orange) and *IPknot* (yellow), **c** Structural Distances of *antaRNA* (blue scaled for different $\mathbb {C}^{\text {gc}}$) and *MODENA* (yellow scaled) using *hotknots* and *IPknot*. For each tool, the targeted pseudoknot categories hairpin (H), bulge (B), complex hairpin (cH) and kissing hairpin (K) are illustrated

Figure 3 provides an overview of the results. For *antaRNA*, results are grouped by the targeted GC-content values $\mathbb {C}^{\text {gc}}$. To identify potential influences on the quality of the design, the data set is grouped according to the declared pseudoknot categories. The performance was compared to the tool *MODENA*. Since *MODENA* supports two different pseudoknot folding prediction tools, namely *IPknot* and *hotknots*, both results are presented.

**GC deviation** ***d***_gc_ The targeted GC-content is precisely produced by *antaRNA*: the GC distance *d*_gc_ is 0 for all GC constraints, as shown in Fig. 3 a. *MODENA* does not provide GC-content driven optimization but shows to have an intrinsic tool dependent GC bias: the GC content of *MODENA* sequences is on average about 55−60 *%* (Fig. 3 b). Noticeable is the fact, that the variance of GC values is wider in low complexity pseudoknot categories (H). The median is slightly lower, when *hotknots* is used.

**Structural Distance** ***d***_str_ Compared to the approach of *MODENA*, *antaRNA* (blue in Fig. 3 c) usually predicts the structure with high accuracy, exhibiting only a small variation among the structure distances within the different pseudoknot categories. The structural distances of *antaRNA* display the growing complexities of the respective pseudoknot categories. While for H- and B-type structures the *d*_str_ median of *antaRNA* is about 0, the medians of cH- and K-type structures do not exceed *d*_str_ of 2.5 *%*. Nevertheless, with increasing structure complexity (H- to K-type), the upper quartiles of the distributions escalate to a *d*_str_ value of 1.5 *%* for B-type, 3 *%* for cH-type and about 7 *%* in the case of K-type structures.

In contrast, *MODENA* (yellow in Fig. 3 c) shows for both predictors (*hotknots* and *IPknot*) *d*_str_-medians between 5 *%* and 12 *%*. Hereby, *hotknots* performs better than *IPknot*, especially in the case of H- and K-type structures. In B-type, the *IPknot* distribution’s lower quartile does not reach 0 but is about 4 *%*. The upper quartiles range from 10 *%* (B-type, *IPknot*) up to 25 % (cH- and K-type, *hotknots*). No correlation of structure’s pseudoknot complexity and the resulting structural distance is visible.

## Discussion

### Performance

As shown in [28] for nested structures, *antaRNA*’s key feature is its reliable design of sequences that show the targeted GC-content. Within this study, we illustrated that this still holds when designing sequences for pseudoknot structure constraints. It was shown that *antaRNA* performs very well in this respect in combination with structure constraints from different pseudoknot structure complexity classes for various targeted GC-content values.

Although the structural distances produced by *antaRNA* grow with increasing pseudoknot complexity (H- via B- and cH- to K-type), *antaRNA* outperforms the current ‘state-of-the-art’ tool *MODENA*. The obtained structural distances for *antaRNA* are about 5−10 *%* lower compared to *MODENA* and additionally show a maximal median structure deviation of 2.5 *%*, depending on the pseudoknot category.

### Optimization strategy

The ant colony optimization strategy applied in *antaRNA* outperforms the strategy applied in *MODENA*. Both tools are heuristics that use external folding prediction programs to evaluate designed sequences. *MODENA* uses a stability and a similarity score to evaluate current solution sequences in order to select parents for offspring generations within its genetic algorithm. *antaRNA* directly uses the specified objectives and shares the information of the currently best solutions in the terrain graph. In this way subsequent ants (i.e. sequence designs) are biased towards the direction of the targeted sequences.

Within the genetic operators of *MODENA*, random crossover and point mutations are introduced into parental sequences. Those mutations are inherited to a child generation. Compared on the structural level, the mutational approach seems less focused in the sense, that good and correct (partial) solutions are only highlighted by being not mutated. In contrast, in *antaRNA* the adaptive local search is capable of promoting good partial solutions in successive runs. This behavior, in combination with a good transmission of current solution qualities into the decision making process of making new solutions, might be the basic reason for *antaRNA*’s advantage in optimizing the problem at hand.

## Conclusion and outlook

Within this study it was shown that *antaRNA*, by incorporating *pKiss* is capable of solving the inverse folding problem for pseudoknot structure constraints under additional side constraints like a targeted GC-content. Currently, the common pseudoknot classes H, B, cH and K are supported. This restriction is inherited from the used *pKiss* mfe-structure predictor. Still, the flexibility of the *antaRNA* framework allows for the integration of even more general pseudoknot structure prediction tools. Due to the immense increase in prediction runtime and only limited increase in applicability, more complex predictors are not wrapped by *antaRNA*.

For known pseudoknot structures, the sequences produced by *antaRNA* show only minor structural deviation of their mfe-structure from the respective targets. While not explicitly shown in the paper, *antaRNA* features a flexible framework to further restrict the sequences produced via the definition of hard and soft sequence constraints. In addition, *antaRNA* introduces precise GC content control to the RNA inverse folding problem of pseudoknot structures, which was not existent before.

In general, besides its good compliance with multi-objective constraints, it was demonstrated that *antaRNA* provides a highly flexible platform to solve the RNA inverse folding problems for pseudoknot structures. It is build in a way that the underlaying routines can be easily adapted and extended to even more complex problems.

## Availability

*antaRNA* is written in Python and available at http://www.bioinf.uni-freiburg.de/Software. Based on the choice if targeting nested or pseudoknot structures, it depends on *RNAfold* or *pKiss*, respectively. Further specifications are listed on the tool’s homepage. *antaRNA* can be additionally found on the Freiburg RNA Tools webserver at http://rna.informatik.uni-freiburg.de including explanations and examples. Links to the Galaxy-RNA-workbench Docker Image and the whole Galaxy Docker Image can also be found on the homepage of *antaRNA*.
